# Subdeltoid Lipoma Associated With Subscapularis Tear Successfully Treated With Arthroscopic Resection and Cuff Repair: A Case Report

**DOI:** 10.7759/cureus.38176

**Published:** 2023-04-26

**Authors:** Sittan Aimprasittichai, Wasaphon Suphakitchanusan, Nithi Pakmanee, Siravich Suvithayasiri, Pichaya Thanindratarn

**Affiliations:** 1 Department of Orthopedics, Chulabhorn Hospital, Bangkok, THA

**Keywords:** shoulder pathology, arthroscopic resection, subscapularis tear, subdeltoid, lipoma

## Abstract

A 61-year-old male patient presented with left shoulder pain and an associated lump. Magnetic resonance imaging revealed a subscapularis tear, and subdeltoid lipoma obliterated its insertion. He was successfully treated with arthroscopic subscapularis repair and resection of mass simultaneously.To the authors' knowledge, this will be the first documented case of lipoma occurring under the deltoid muscle associated with the subscapularis tear. The reported arthroscopic approach for resection of the subdeltoid lipoma provides a complete removal, minimal muscle dissection, limited surgical scar, and satisfying functional outcomes. Therefore, it may be considered an option for benign tumor resection in this area.

## Introduction

Isolated subscapularis tear is a relatively uncommon injury, especially when compared with tears of the supraspinatus and infraspinatus muscles [1]. Mechanical contact between the coracoid and lesser tuberosity, which is suggested to impinge the tendon, is considered one of the causes of a subscapularis tear [2-4]. In some cases, growing lesions, such as tumors, have been reported to obliterate the subcoracoid space and cause subcoracoid impingement and subsequent subscapularis tear [5,6].

Lipomas are the most common benign soft tissue tumors in the shoulder region, which are most commonly located in subcutaneous tissue [7]. There have been few reports of intramuscular lipoma within supraspinatus causing subacromial impingement [8-11]. To our knowledge, there have been no previous reports of lipomas in the subdeltoid space causing subscapularis tears.

Open resection with marginal excision is the classic surgical approach for lipoma resection [12,13]. Arthroscopic marginal resection of intramuscular lipoma in the shoulder area has also been reported but focusing mainly on lipomas within the supraspinatus muscle [8,9].

In this case report, we present the rare case of a lipoma within the subdeltoid space that was associated with a subscapularis tear. The patient was successfully treated with arthroscopic resection of the lipoma and rotator cuff repair.

## Case presentation

Case description

**Figure 1:**
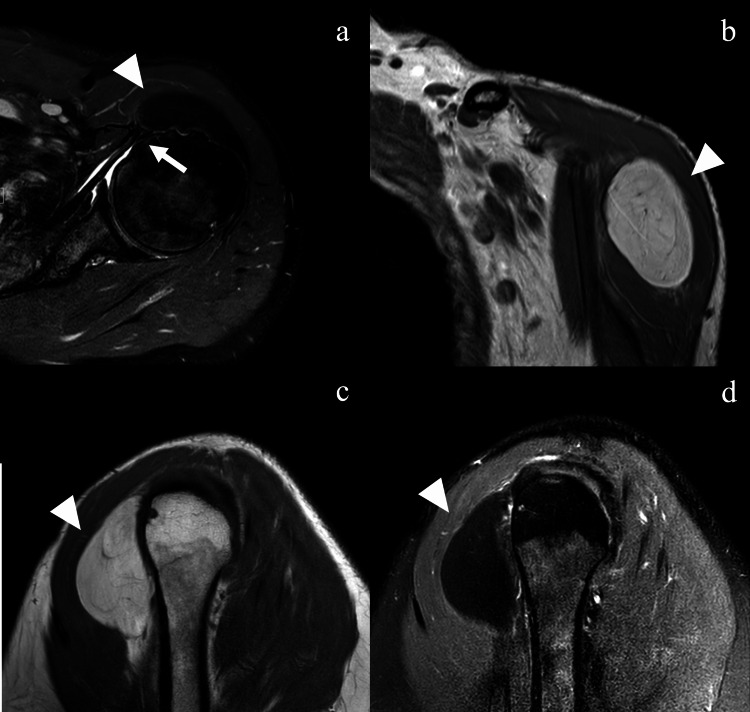
Preoperative magnetic resonance imaging Preoperative axial MRI show mass obliterated anterior aspect of lesser tuberosity (a, arrowhead) with full thickness tear of the subscapularis tendon and subluxation of the long head biceps tendon (a, arrow). The oblique coronal and sagittal MRI show a 46x35x48 mm homogeneous tumor under the deltoid muscle (b and c, arrowhead). The tumor appears signal-hyperintense on T1-weighted images (a and c) and suppressed on T2- weighted fat-suppression images (d).

A 61-year-old male patient presented with left shoulder pain. The pain was gradually worse. He felt he was unable to lift the heavy object as usual. The shoulder examination findings revealed the limited extreme elevation of the shoulder due to pain. Neer and Hawkins' impingement tests were positive. The Jobe test was negative for weakness but aggravated some pain. The belly press, bear hug, and lift-off tests were all positive. There was a 5 cm soft lump at the anterolateral aspect of the shoulder with no tenderness and a smooth surface by palpation. The neurovascular status of his upper extremity was unremarkable. The symptoms were not improved after analgesic drugs and physiotherapy over a period of three months. Magnetic resonance imaging (MRI) showed a mass obliterated anterior aspect of lesser tuberosity with full thickness tear of the subscapularis tendon and subluxation of the long head biceps tendon (Figure 1a). A mass was well-circumscribed and located in a plane deep to the deltoid muscle. The lesion signal was bright on T1-weighted images that were suppressed with the fat-suppression technique and had a dark appearance on T2-weighted images that were consistent with fatty tissue (Figures 1b-c). The lesion was approximately 46x35x48 mm. The radiologist concluded that the mass was most likely a soft-tissue lipoma.

Surgical technique

**Figure 2:**
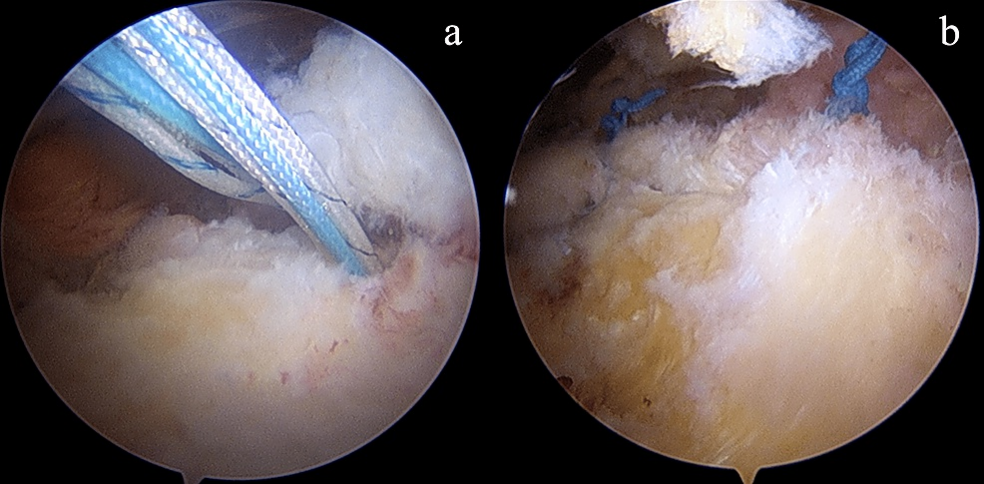
Subscapularis tear Arthroscopic images show subscapularis tendon repair. Two 4.5 mm double-loaded soft suture anchors were inserted through the medial border of the subscapularis footprint on lessor tuberosity (a). The subscapularis tendon was repaired with a single row anchor suture in a modified Mason-Allen configuration (b).

**Figure 3:**
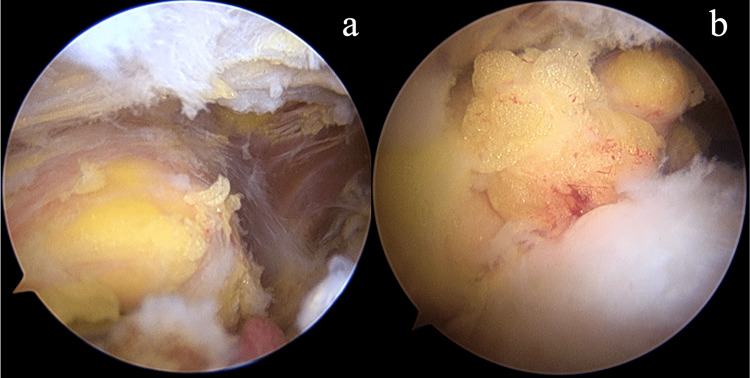
Subdeltoid lipoma Arthroscopic images show encapsulated fat-containing globule beneath the deltoid fiber and attached to the lateral portion of the transverse ligament with fibrous adhesion (a, b).

**Figure 4:**
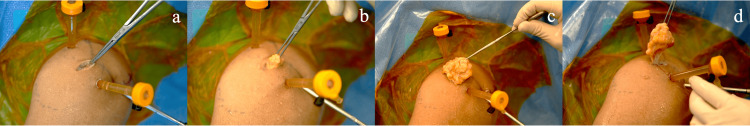
Removal of lipoma Intraoperative images show a yellowish lipomatous mass is resected and removed with an Allis forceps through a 2.5 cm extended superolateral portal (a, b, c, and d).

**Figure 5:**
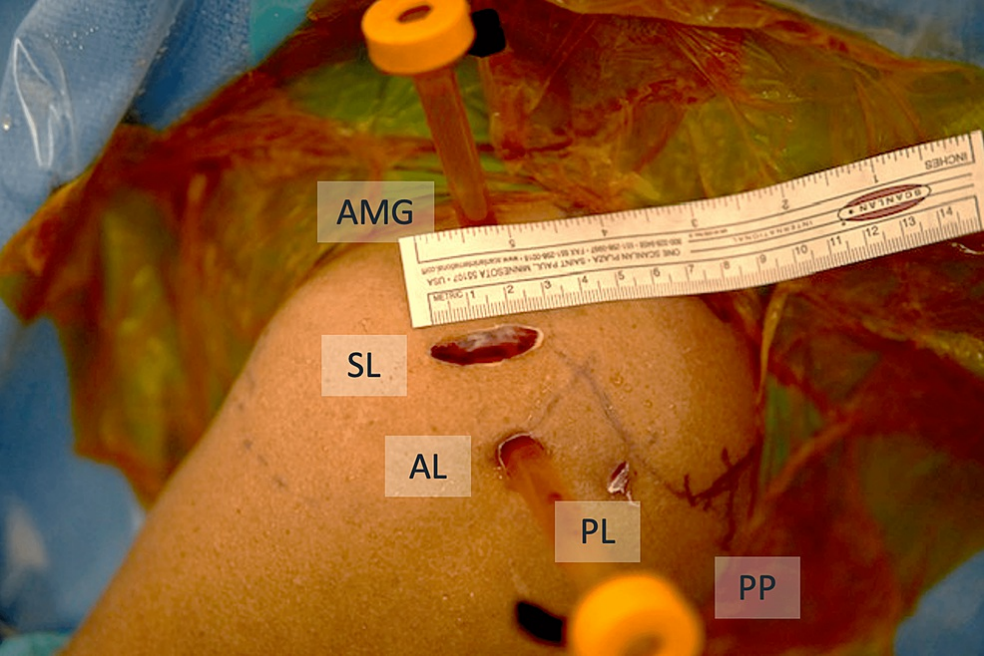
Arthroscopic portals Photograph taken during the resection shows the five portals established for the procedure. The first portal is a standard posterior portal (PP) located 3 cm inferior and 2 cm medial to the posterolateral edge of the acromial process. The second portal, the posterolateral portal (PL), located at the lateral to the posterior one-third of the acromial border, serves primarily as a viewing portal for the resection process. The third portal, the anterolateral portal (AL), located 2 cm below the anterior one-third of the acromial border, is used for instrumentation. The fourth portal, an anterior mid-glenoid portal (AMG) established just lateral to the tip of the coracoid process, is used for anchor insertion, knot tying for the subscapularis repair, and instrumentation during the resection. The fifth portal, the 2.5 cm extended superolateral portal (SL), serves as the removal channel located at the edge of the anterolateral aspect of the acromion process.

The patient was placed in the beach-chair position after general anesthesia combined with an interscalene block. The 30° arthroscope was introduced through the standard posterior portal. We performed a standard arthroscopic examination of the glenohumeral joint. The anterior mid-glenoid portal was established just lateral to the tip of the coracoid process through the rotator interval as the working instrument portal. The long head of the bicep tendon was tenotomized. The torn subscapularis tendon and the footprint were accessed and then debrided by the motorized shaver. Then, we moved the arthroscope to the subacromial space. The third portal, which is known as the anterolateral portal, was created laterally to the anterior one-third of the acromial border and served as the working portal for the routine subacromial decompression procedure. The fourth portal, the posterolateral portal, was created at the lateral to the posterior one-third of the acromial border for switching the viewing portal from posterior to lateral to obtain better vision. Two 4.5 mm double-loaded soft suture anchors were introduced via the anterior mid-glenoid portal and inserted through the medial border of the subscapularis footprint on lessor tuberosity. All sutures were passed through the tendinous part of the subscapularis tendon by the suture passer via the anterolateral portal in the modified Mason-Allen configuration. The superior and inferior limbs from the inferior anchor were tied first, and the two other limbs from the same anchor were tied consecutively. The sutures from the superior anchor were tied in the same fashion (Figures 2a-b). After the repair, we accessed the subdeltoid space by the plane connecting from the subacromial space. We found a large encapsulated fat-containing globule beneath the deltoid fiber attached to the lateral portion of the transverse ligament with fibrous adhesion (Figures 3a-b). The superolateral portal was created at the edge of the anterolateral aspect of the acromion process, which was located just superior to the lipoma. On blunt dissection, we used the Allis forceps to grasp the lipoma via the superolateral portal. The peanut sponges and the radiofrequency probe were introduced through the anterolateral portal to bluntly dissected the surrounding adhesion circumferentially. The bleeding was stopped by the radiofrequency probe before the removal. The superolateral portal was extended to 2.5 cm. The mass was finally excised and removed via the extended superolateral portal (Figures 4-5).

**Figure 6:**
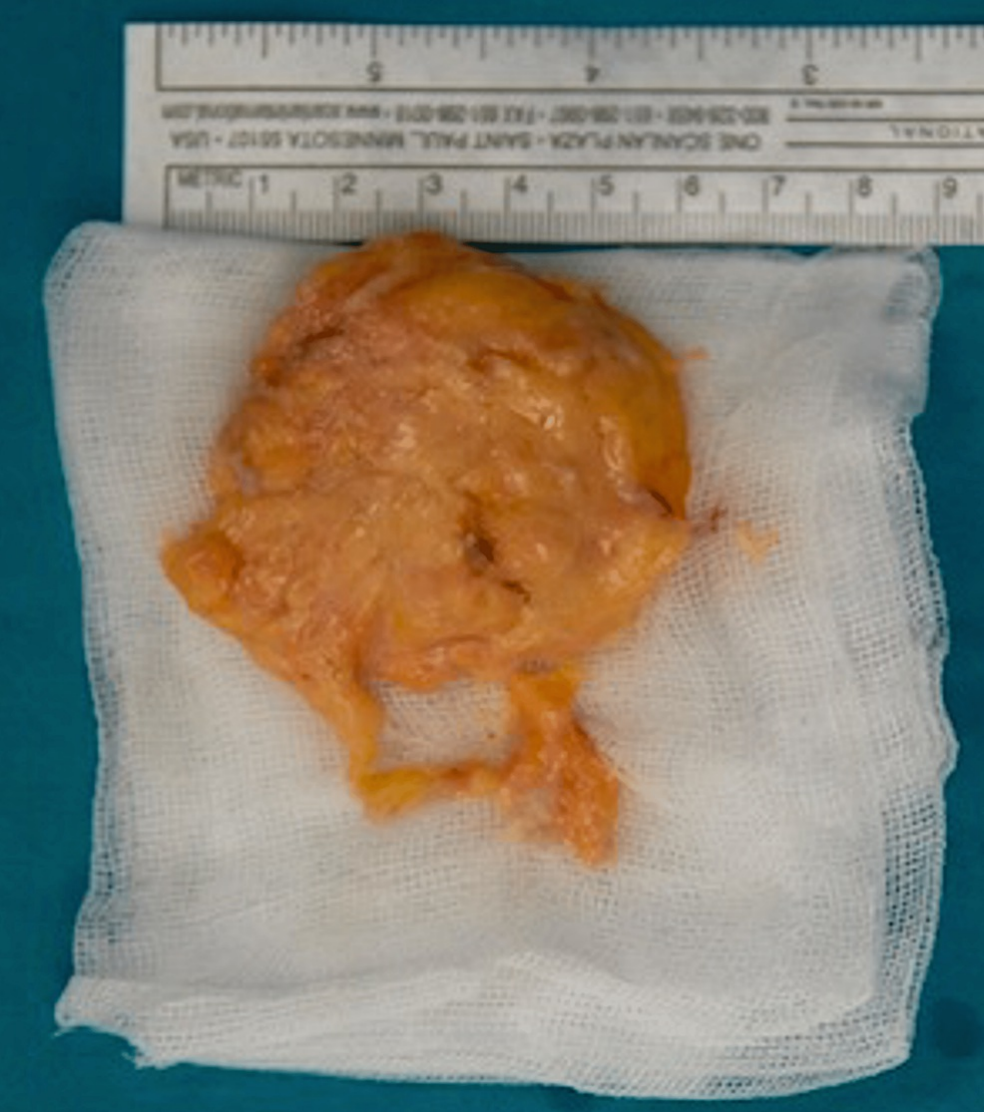
Gross view of the resected tumor The intraoperative image shows a gross view of the marginal resected tumor. The mass was well-circumscribed with a fatty appearance.

The macroscopic specimen is shown in Figure 6. The mass was submitted for histopathologic examination, and the result showed a benign lipoma. The patient was discharged on the first postoperative day with a shoulder immobilizer (Ultrasling; Donjoy, Vista, CA, USA) for four postoperative weeks. During the six weeks following the immobilization period, the patient was permitted to increase the range of motion progressively. At postoperative 12-20 weeks, a strengthening exercise program was permitted.

Clinical outcomes

He recovered well and resumed activities of daily living and work without pain after the postoperative six months. Follow-up MRI performed at postoperative six months showed a healed rotator cuff tendon without retear and complete lipoma removal without gross residual tumor. The visual analogue scale score was one out of 10, and the Constant-Murley score was 89 at one-year postoperative.

## Discussion

To our knowledge, this is the first report of intramuscular lipoma in subdeltoid space associated with subscapularis tear. Lipoma is one of the most common soft-tissue tumors; it is predominantly located in the subcutaneous tissues [14]. However, there are few reports that intramuscular lipoma in the supraspinatus muscle could be associated with subacromial impingement due to the thickening of the supraspinatus muscle [8-11]. In our case, the tumor was located adjacent to the subscapularis insertion, and the growing mass in this limited space could have compressed the subscapularis tendon, causing coracoid impingement and ultimately leading to the subscapularis tear.

The subscapularis muscle is the largest and strongest of the rotator cuff muscles and is responsible for the internal rotation of the shoulder joint [15]. Subscapularis tears are less common than supraspinatus tears but can occur due to trauma or degenerative changes associated with subcoracoid impingement [1]. In this case, the subscapularis tear was found to be associated with the adjacent intramuscular lipoma in the subdeltoid space, which may have contributed to the subcoracoid impingement and compression of the subscapularis tendon. The compressive force from the growing lipoma could have led to irritation and inflammation of the subscapularis tendon, ultimately leading to a tear. While this mechanism of injury is plausible, further research is necessary to confirm the relationship between intramuscular lipomas and subscapularis tears. It is important to consider this potential cause when evaluating patients with subscapularis tears, especially if they have a concurrent mass in the subdeltoid space.

In our case, the anticipated tumor size was 46x35x48 mm. Because the tumor size <50 mm in diameter is likely benign [16], we decided to perform marginal resection without biopsy. In cases of subdeltoid lipomas, an open resection has traditionally been considered the treatment of choice [12,13]. However, with an advance in arthroscopic techniques, the subdeltoid space can be assessed by arthroscopy. There is limited literature available on the association between intramuscular lipomas in the subdeltoid space and rotator cuff injury, as this is a rare presentation, and all of them reported about supraspinatus tear. Conesa et al. in 2015 reported a case of a patient who presented with a large intramuscular lipoma in the subdeltoid space, which compressed the supraspinatus muscle and resulted in shoulder impingement syndrome which was successfully treated with arthroscopic removal. The authors concluded that arthroscopic removal is less invasive and shows similar results to conventional open surgery [9]. In addition, a case report by Nakamura et al. published in 2021 described a patient who presented with a case of lipoma under the supraspinatus muscle, which was located in the supraspinatus fossa where the suprascapular nerve runs. The authors reported successful arthroscopic resection of the lipoma combined with suprascapular nerve release [8]. In our case, the lipoma occurred deep to the deltoid muscle, concomitant with a subscapularis tear. We performed an arthroscopic resection combined with rotator cuff repair, which successfully relieved all clinical symptoms. While the arthroscopic technique may be time-consuming, it provides several advantages, including minimal muscle dissection, better visualization, small incisions, better cosmetic outcomes, and the ability to manage rotator cuff pathology simultaneously.

However, some limitations of our study should be acknowledged. Firstly, this was a single case report, and further studies are needed to confirm the efficacy of arthroscopic resection for subdeltoid lipomas in a larger population. Secondly, the mechanism of injury described in this case report is hypothetical, and more research is needed to confirm the relationship between intramuscular lipomas and subscapularis tears. Finally, the follow-up period in this case report was limited to one year, and longer-term outcomes following arthroscopic resection for subdeltoid lipomas remain unknown.

## Conclusions

This case report highlights the rare occurrence of an intramuscular lipoma in the subdeltoid space associated with the subscapularis tear. While most lipomas are located in subcutaneous tissues, this case demonstrates that growing masses in limited spaces may compress adjacent structures, leading to injury. The use of arthroscopic resection combined with rotator cuff repair proved to be a safe and effective treatment option for this benign tumor. Arthroscopic resection offers minimal muscle dissection, better visualization, small incisions, and a better cosmetic outcome. Therefore, it can be considered a viable and minimally invasive treatment option for benign tumors in the subdeltoid space. Further studies are necessary to confirm this mechanism of injury.
